# Workaholism as a Risk Factor for Depressive Mood, Disabling Back Pain, and Sickness Absence

**DOI:** 10.1371/journal.pone.0075140

**Published:** 2013-09-25

**Authors:** Ko Matsudaira, Akihito Shimazu, Tomoko Fujii, Kazumi Kubota, Takayuki Sawada, Norimasa Kikuchi, Masaya Takahashi

**Affiliations:** 1 Clinical Research Center for Occupational Musculoskeletal Disorders, Kanto Rosai Hospital, Kawasaki City, Kanagawa, Japan; 2 Department of Mental Health, Graduate School of Medicine, The University of Tokyo, Bunkyo-ku, Tokyo, Japan; 3 CLINICAL STUDY SUPPORT, Inc., Nagoya City, Aichi, Japan; 4 National Institute of Occupational Safety and Health, Kawasaki City, Kanagawa, Japan; Catholic University of Sacred Heart of Rome, Italy

## Abstract

**Objectives:**

Although it is understood that work-related factors, including job demands, job control, and workplace support, are associated with workers' health and well-being, the role played by personal characteristics, especially workaholism, has not been fully investigated. This study examined workaholism's associations with psychological ill health, low back pain with disability, and sickness absence among Japanese workers.

**Methods:**

A cross-sectional Internet survey was conducted using self-administered questionnaires. Data from 3,899 Japanese workers were analyzed. Workaholism was measured using the Dutch Workaholism Scale (DUWAS). Scores were divided into tertiles, where respondents were classified into three groups (high, middle, and low). Depressive mood as a measure of psychological ill health was assessed using the SF-36 mental health subscale, and low back pain using a standardized question. Sickness absence, except that due to physical injuries, was categorized either as absence due to mental health problems or to physical/somatic problems including the common cold. Multiple logistic regression analyses were conducted to examine the association between workaholism and depressive mood, low back pain with disability, and sickness absence, adjusting for demographic characteristics, job demand, job control, and workplace support.

**Results:**

Compared to the low workaholism group, the middle and high workaholism groups had significantly higher odds for depressive mood (Odds ratio (OR) = 1.93 and 3.62 for the middle and high groups, respectively), disabling back pain (ORs = 1.36 and 1.77 for the middle and high groups, respectively). Workaholism was more strongly associated with sickness absence due to mental health problems than that for other reasons (ORs = 1.76 vs. 1.21 for the middle group and 3.52 vs. 1.37 for the high groups).

**Conclusions:**

Workaholism is significantly associated with poor psychological health, disabling back pain, and sickness absence, particularly from mental health problems. Therefore, workaholism must be considered when addressing well-being of workers.

## Introduction

The impact of job strain on the physical and psychological well-being of workers has been extensively studied with regard to job characteristics [1]–[5]. However, while the costs associated with fulfilling job demands result in adverse health outcomes, workers' personal characteristics may also affect these outcomes [6]. One personal characteristic associated with workers' well-being is workaholism [7]. Its definition comprises two dimensions: the tendency to work excessively hard (the behavioral dimension), and an obsession with work (the cognitive dimension). These behaviors manifest as compulsive working [7]. Scott et al. proposed the following three fundamental characteristics of workaholics: (1) they spend a great deal of time on work activities when given the discretion to do so, which results in giving up important social, family, or recreational activities because of work; (2) they persistently and frequently think about work when not at work; and (3) they work beyond what is reasonably expected to meet requirements of either the job or their basic economic needs [8]. A similar construct, Type A personality, can lead to work addiction; however, Type A personality represents cognitions and behaviors in terms of overall life situations, rather than work-specific situations as is the case with workaholism [9].

Many studies have reported an association between workaholism and psychological well-being (i.e., psychological distress, lowered emotional well-being, exhaustion, and sleep problems) [6], [7], [10]–[12]. Workaholic people spend an excessive amount of time at work, and this may leave them without sufficient opportunities to recover from such excessive efforts. Over time, this leads to emotional exhaustion [6], [13]. The persistence and frequency with which they think about work [6] may also result in sympathetic arousal and emotional distress [14]. In addition, workaholic people tend to display a higher degree of perfectionism and an unwillingness to delegate tasks, which may lead to hostile interpersonal relationships and/or ineffective team membership [8], [15]. These characteristics of workaholics may explain the association between workaholism and poor mental health.

Studies examining the association between workaholism and overall physical well-being have reported that workaholism is positively associated with physical complaints [7], [10], [11], [16]. However, associations between workaholism and specific physical complaints such as low back pain (LBP) have not been examined. LBP is a common major health problem in both Western countries and Japan [17]. It is a major cause of disability and work-related losses, resulting in both direct and indirect social costs [18]–[21]. Although the majority of individuals seeking help for LBP have low-grade LBP with low disability [22], [23], some individuals develop chronic and disabling back pain. The Global Burden of Disease (GBD) studies done in 1990 and 2000 have demonstrated that low back pain is one of the major specific causes associated with years living with disability (years of life lived in less than ideal health) [24]. The costs associated with chronic LBP far exceed those for acute LBP [25]. The association between psychosocial factors and back pain outcomes has been studied for decades, but most of these studies have focused on work-related factors, including heavy physical work demands, poor relations with colleagues, and job class [26]–[28]. However, to our knowledge, no previous study has examined the association between LBP and workaholism.

Given the association between workaholism and poor psychological/physical health, it seems reasonable to assume that workaholism is also associated with sickness absence (a behavioral aspect of ill health). However, because workaholics have difficulty detaching from work and delegating tasks to others (preventing these individuals from missing work), it is also possible to assume that workaholism is associated with less sickness-related absence [15]. To date, only one study has examined the association between workaholism and sickness absence. Burke and Matthiesen reported no significant difference between workaholics and non-workaholics in the number of workdays absent [29]. However, their sample group consisted only of journalists. Therefore, it is appropriate to examine a variety of occupational groups in order to examine whether these results are applicable to other occupations. In addition, we need to test if the workaholics would have more sickness absence from mental health problems compared to other problems.

Against this background, the aim of the present study was to examine the associations between workaholism on the one hand and psychological ill health, low back pain with disability, and sickness absence on the other among Japanese workers.

## Methods

### Ethics Statement

This study was approved by the medical/ethics review board of the Japan Labour Health and Welfare Organization.

### Study population

An Internet research company with 1.5 million registered research volunteers, aged 20–69 years, was used to conduct an Internet-based survey on occupation and health, in 2011. We randomly selected 106,250 volunteers from 201,170 monitors, living in three greater metropolitan areas of Japan (23 wards of Tokyo, the City of Osaka, and the City of Nagoya). On March 25, 2011, the selected volunteers were invited to take part in the study via an e-mail containing a link to the survey. Participants received online shopping points as an incentive for participation. In order to prevent double registration, e-mail addresses were checked and a link to the questionnaire was disabled once the survey was completed. On March 31, 2011, the survey was closed when more than 5 thousand participants responded (a total of 5,917 surveys were collected). Therefore, a specific response rate was not relevant to this survey. The proportion of respondents working within primary (e.g., agriculture, forestry, and fisheries) and secondary (e.g., mining, manufacturing, and construction) industries was extremely low (0.7% and 7.5% respectively). Therefore, we analyzed responses only from those individuals working in the tertiary industry (e.g., transport and postal activity, wholesale and retail trade, accommodations, eating and drinking services, finance and insurance, advertising, education and learning support, and medical, health care and welfare). Individuals with a reported age of either ≤20 years or >65 years, those who did not report their occupational category, and those who had worked for less than one year were excluded. A total of 3,899 participants were retained and included in the analysis.

### Demographic characteristics

The questionnaire included demographic information, such as age, sex, weight, height, smoking habits, highest education attained, regular exercise, whether more than half the working hours involved repetitive physical activity (blue-collar work), and present illness. The definition of regular exercise was based on that provided by the National Health and Nutrition Survey by Japan's Ministry of Health, Labour and Welfare [30]. Regular exercise was considered to be physical exercise performed more than twice a week, for 30 min or longer, for more than one year.

### Workaholism

Workaholism was measured using the Dutch Workaholism Scale (DUWAS) [13]. This scale consists of two subscales: work excessively (WE) and work compulsively (WC). Each subscale consists of 5 items rated on a 4-point Likert scale ranging from 1 =  *totally disagree* to 4 =  *totally agree*. Examples of the items used are “I seem to be in a hurry and racing against the clock” and “I feel that there's something inside me that drives me to work hard.” Both subscales had good reliability (Cronbach's alpha = 0.81 and 0.77 for WE and WC respectively). Respondents were classified intro three groups on the basis of which tertile of the total workaholism score they fell into (high, middle, and low) [31].

### Work-related variables

Weekly work hours were measured with a set of response options (<40, 40–45, 45–50, 50–55, 55–60, 60–65, and 65 or more hours per week).A subscale of the Brief Job Stress Questionnaire [32] was used to measure job demands, job control, and workplace support. Each item was rated on a 4-point Likert scale ranging from 1(*strongly disagree*) to 4 (*strongly agree*). Job demand was calculated by summing the item scores for psychological job overload (six items). Job control was calculated by summing the item scores for job control (three items). Workplace support was calculated by summing the item scores for supervisor support (three items) and coworker support (three items). The Cronbach's alpha coefficients were 0.83 for psychological job demand, 0.76 for job control, and 0.88 for workplace support. Respondents were classified into three groups each for those variables on the basis of the tertile within which their scores fell (high, middle, and low).

### Outcome variables

The following three outcomes were examined: psychological ill health, LBP with disability (disabling back pain), and sickness absence. Mental health was measured using the SF-36 version 1.2 [33]–[35]. Psychological ill health was assessed using a SF-36 mental health summary score; depressive mood was determined if the summary score was 52 or lower (based on a general cut-off for Japanese) [36]. LBP was defined as pain in the area between the lower costal margin and the gluteal folds; lasting more than one day; occurring regardless of accompanying radiating pain; and not associated with merely febrile illness, menstrual periods, or pregnancy [37]. Respondents were given a diagram with a shaded area to illustrate the area of LBP. Disabling back pain was defined as LBP that had occurred within one year and caused disruption to a worker's job, regardless of whether this had resulted in an absence from work. Sickness absence was defined as any absence due to sickness except the physical injuries such as fractures and sprains within one year. We separated sickness absence due to mental health problems (e.g., depression, panic disorder, and autonomic ataxia) from absence due to other reasons (e.g., neck and back pain, headache, gastrointestinal discomfort, excessive fatigue, and the common cold).

### Statistical analysis

Initially, a simple descriptive analysis was conducted. This was followed by a separate logistic regression examining the associations between workaholism and the three outcomes (depressive mood, disabling back pain, and sickness absence). First, a univariate analysis of the association between workaholism and each outcome was conducted. Second, the association between workaholism and each outcome was examined using a multiple logistic regression adjusted for demographic characteristics, including age, gender, being overweight, smoking habits, educational level, regular exercise, work status, and present illness. Third, the association between workaholism and each outcome was examined using multiple logistic regression adjusted for demographic characteristics and work-related variables (weekly work hours [<60 vs. ≥60 h], job demand, job control, and workplace support). Participant age was categorized into three groups: 20–39, 40–49, and 50–64 years. Body Mass Index (BMI) was calculated using self-reported weight and height. “Overweight” was defined as having a BMI of 25 or higher. Smoking status was dichotomized into “current smoker” and “non-current smoker” (non-smoker and ex-smoker). Educational level was also dichotomized into “non-college” and “college or over.” Work status was classified as “blue collar worker” and “other workers.” The low workaholism group was used as a reference category, for which an odds ratio (OR) for the univariate analysis, adjusted odds ratios (aOR) for multivariate analyses and 95% confidence intervals (95% CI) were calculated. Statistical analyses were conducted using SAS 9.3 (SAS Institute Inc., Cary, NC). All statistical tests were two-tailed with a significance level of 0.05.

## Results

### Characteristics of participants

Participants' characteristics are shown in Table 1. Their mean age was 45 years (SD = 11.8). Of the 3,899 participants, 50.7% were men and 57.1% had bachelor's degree or above. Participant occupations were as follows: professional or technicians (35.2%), clerks (26.8%), sales workers (12.8%), service workers (11.8%), managers (9.2%), transportation or communications workers (3.2%), and security workers (1.2%). Those who worked 60 or more hours per week was 10.8%. Characteristics of the participants according to the three categories of workaholism are summarized in Table 2. Overall, individuals with higher workaholism were more likely to be younger, current smokers, blue-collars, and to work longer. They also reported high job demand, low job control, and low social support at work.

**Table 1:** Sample characteristics (N = 3,899).

	n (%)
Age (yr), mean (SD)	45.0 (11.8)
Age	
20–39	1,447 (37.1)
40–49	898 (23.0)
50–64	1,554 (39.9)
Men	1,976 (50.7)
Overweight	833 (21.4)
Current smoker	1,010 (25.9)
Education	
College−	1,664 (42.7)
College+	2,226 (57.1)
Other	9 (0.2)
Regular exercise	931 (23.9)
Blue-collar	579 (14.9)
Present illness	1,100 (28.2)
Work hours (per week)	
<60 h/wk	3479 (89.2)
≥60 h/wk	420 (10.8)
Job classification	
Professional or technician	1,373 (35.2)
Managers	357 (9.2)
Clerk	1,043 (26.8)
Sales worker	500 (12.8)
Service worker	458 (11.8)
Security worker	45 (1.2)
Transportation or communications worker	123 (3.2)
Workaholism score, mean (SD)	18.7 (6.2)
Job demand, mean (SD)	14.2 (3.9)
Job control, mean (SD)	6.8 (2.2)
Workplace support, mean (SD)	14.8 (4.4)

SD, standard deviation.

**Table 2:** Demographic variables by workaholism (N = 3,899).

Variables, n (%)	Workaholism			p-value*
	Low (n = 1,372)	Middle (n = 1,396)	High (n = 1,131)	
Age				0.001
20–39	465 (33.9)	522 (37.4)	460 (40.7)	
40–49	307 (22.4)	325 (23.3)	266 (23.5)	
50–64	600 (43.7)	549 (39.3)	405 (35.8)	
Men	670 (48.8)	717 (51.4)	589 (52.1)	0.222
Overweight	287 (20.9)	309 (22.1)	237 (21.0)	0.681
Current smoker	321 (23.4)	358 (25.6)	331 (29.3)	0.004
College+	798 (58.3)	798 (57.3)	630 (55.9)	0.459
Regular exercise	336 (24.5)	348 (24.9)	247 (21.8)	0.156
Blue-collar	162 (11.8)	234 (16.8)	183 (16.2)	<0.001
Present illness	367 (26.8)	406 (29.1)	327 (28.9)	0.325
Work hours (per week) ≥60 h/wk	54 (3.9)	125 (9.0)	241 (21.3)	<0.001
Job demand				<0.001
Low	635 (46.3)	257 (18.4)	86 (7.6)	
Middle	550 (40.1)	656 (47.0)	361 (31.9)	
High	187 (13.6)	483 (34.6)	684 (60.5)	
Job control				<0.001
High	735 (53.6)	674 (48.3)	495 (43.8)	
Middle	388 (28.3)	425 (30.4)	356 (31.5)	
Low	249 (18.2)	297 (21.3)	280 (24.8)	
Workplace support				0.002
High	526 (38.3)	559 (40.4)	367 (32.5)	
Middle	464 (33.8)	458 (32.8)	416 (36.8)	
Low	382 (27.8)	379 (27.2)	348 (30.8)	

*P-values were calculated by Chi-square test.

### Depressive mood

As per the SF-36 mental health score, 33.9% of the respondents had depressive symptoms. The association between workaholism groups and depressive mood is shown in Table 3. In the unadjusted model, the middle and high workaholism groups had significantly higher odds for depressive mood compared with the low workaholism group (ORs = 2.02 and 4.25 for the middle and high groups, respectively). In addition, the model adjusted for demographics also showed significantly higher odds for depressive mood in these groups compared with the low workaholism group (aORs = 1.98 and 4.17 for the middle and high groups, respectively). Furthermore, in the fully adjusted model, these groups continued to show significantly higher odds for depressive mood compared with the low workaholism group (aORs = 1.93 and 3.62 for the middle and high groups, respectively).

**Table 3:** Association between workaholism and depressive mood (N = 3,899).

	OR	(95% CI)
**Unadjusted model**		
Workaholism		
Low	1.00	
Middle	2.02	(1.70–2.41)
High	4.25	(3.56–5.07)
**Demographic adjusted model**		
Workaholism		
Low	1.00	
Middle	1.98	(1.67–2.36)
High	4.17	(3.48–4.98)
Age		
20–39	1.00	
40–49	0.84	(0.70–1.01)
50–64	0.59	(0.50–0.70)
Women vs. men	1.01	(0.87–1.17)
Overweight vs. normal weight	0.96	(0.81–1.15)
Current smoker vs. non-current smoker	1.06	(0.90–1.25)
College− vs. college +	0.98	(0.85–1.13)
Exercise− vs. +	1.34	(1.13–1.58)
Blue collar vs. non blue collar	0.86	(0.70–1.05)
Present illness− vs. +	1.62	(1.38–1.89)
**Fully adjusted model**		
Workaholism		
Low	1.00	
Middle	1.93	(1.60–2.33)
High	3.62	(2.94–4.40)
Age		
20–39	1.00	
40–49	0.83	(0.69–1.00)
50–64	0.61	(0.51–0.72)
Women vs. men	1.03	(0.88–1.20)
Overweight vs. normal weight	0.96	(0.80–1.15)
Current smoker vs. non-current smoker	1.12	(0.95–1.32)
College− vs. college +	0.99	(0.85–1.14)
Exercise− vs. +	1.31	(1.10–1.56)
Blue collar vs. non blue collar	0.84	(0.69–1.04)
Present illness− vs. +	1.63	(1.39–1.92)
Work hours <60 vs. ≥60 h/wk	0.95	(0.75–1.20)
Job demand		
Low	1.00	
Middle	1.13	(0.93–1.38)
High	1.50	(1.27–1.78)
Job control		
High	1.00	
Middle	1.19	(1.00–1.41)
Low	1.64	(1.36–1.98)
Workplace support		
High	1.00	
Middle	1.47	(1.23–1.75)
Low	2.64	(2.20–3.17)

OR: Odds Ratio; CI: Confidence Interval.

### Disabling back pain

Those who reported disabling back pain within the past year were 8.2%. The association between workaholism groups and disabling back pain is shown in Table 4. In the unadjusted model, the middle and high workaholism groups had significantly higher odds for disabling back pain compared with the low workaholism group (ORs = 1.57 and 2.41 for the middle and high groups, respectively). In addition, the model adjusted for demographics also showed significantly higher odds for disabling back pain in these groups compared with the low workaholism group (aORs = 1.50 and 2.33 for the middle and high groups, respectively). These results were consistent with those from the fully adjusted model (aORs = 1.36 and 1.77 for the middle and high groups, respectively).

**Table 4:** Association between workaholism and disabling back pain (N = 3,899).

	OR	(95% CI)
**Unadjusted model**		
Workaholism		
Low	1.00	
Middle	1.57	(1.16–2.13)
High	2.41	(1.79–3.24)
**Demographic adjusted model**		
Workaholism		
Low	1.00	
Middle	1.50	(1.11–2.04)
High	2.33	(1.72–3.14)
Age		
20–39	1.00	
40–49	1.04	(0.76–1.43)
50–64	0.99	(0.75–1.31)
Women vs. men	1.00	(0.79–1.27)
Overweight vs. normal weight	1.05	(0.79–1.39)
Current smoker vs. non-current smoker	1.11	(0.86–1.45)
College− vs. college +	1.13	(0.89–1.44)
Exercise− vs. +	1.14	(0.86–1.51)
Blue collar vs. non blue collar	1.51	(1.12–2.03)
Present illness− vs. +	2.15	(1.69–2.74)
**Fully adjusted model**		
Workaholism		
Low	1.00	
Middle	1.36	(0.98–1.87)
High	1.77	(1.26–2.48)
Age		
20–39	1.00	
40–49	1.04	(0.76–1.42)
50–64	1.00	(0.76–1.33)
Women vs. men	1.11	(0.86–1.42)
Overweight vs. normal weight	1.02	(0.77–1.36)
Current smoker vs. non-current smoker	1.11	(0.85–1.44)
College− vs. college +	1.14	(0.90–1.45)
Exercise− vs. +	1.13	(0.85–1.49)
Blue collar vs. non blue collar	1.52	(1.12–2.05)
Present illness− vs. +	2.18	(1.71–2.78)
Work hours <60 vs. ≥60 h/wk	1.57	(1.12–2.19)
Job demand		
Low	1.00	
Middle	0.93	(0.66–1.32)
High	1.37	(1.04–1.80)
Job control		
High	1.00	
Middle	0.98	(0.74–1.30)
Low	0.99	(0.72–1.34)
Workplace support		
High	1.00	
Middle	1.05	(0.78–1.41)
Low	1.54	(1.15–2.06)

OR: Odds Ratio; CI: Confidence Interval.

### Sickness absence

According to responses, 44.2% reported an absence from work. The association between workaholism groups and sickness absence is shown in Table 5. In the unadjusted model, the middle and higher workaholism groups had significantly higher odds for sickness absence compared with the low workaholism group (aORs = 1.26 and 1.47 for the middle and high groups, respectively). In addition, the model adjusted for demographics also showed significantly higher odds for sickness absence in these groups compared with the low workaholism group (aORs = 1.23 and 1.40 for the middle and high groups, respectively). Furthermore, in the fully adjusted model, these groups continued to show significantly higher odds for sickness absence compared with the low workaholism group (aORs = 1.19 and 1.38 for the middle and high groups, respectively). When limited to sickness absence due to mental health problems, the significant aORs increased to 1.76 for the middle group and to 3.52 for the high group.

**Table 5:** Association between workaholism and sickness absence except that due to physical injuries (N = 3,899).

	Absence (all causes)		Absence (mental problem)		Absence (other causes)	
	OR	(95% CI)	OR	(95% CI)	OR	(95% CI)
**Unadjusted model**						
Workaholism						
Low	1.00		1.00		1.00	
Middle	1.26	(1.08–1.46)	1.87	(1.24–2.82)	1.27	(1.09–1.47)
High	1.47	(1.25–1.72)	3.88	(2.64–5.71)	1.44	(1.23–1.69)
**Demographic adjusted model**						
Workaholism						
Low	1.00		1.00		1.00	
Middle	1.23	(1.05–1.43)	1.74	(1.14–2.64)	1.24	(1.06–1.45)
High	1.40	(1.19–1.65)	3.62	(2.44–5.38)	1.38	(1.17–1.63)
Age						
20–39	1.00		1.00		1.00	
40–49	0.57	(0.48–0.68)	0.57	(0.39–0.83)	0.57	(0.48–0.68)
50–64	0.41	(0.35–0.47)	0.38	(0.27–0.54)	0.42	(0.36–0.49)
Women vs. men	1.19	(1.04–1.36)	1.08	(0.80–1.47)	1.21	(1.06–1.39)
Overweight vs. normal weight	1.19	(1.01–1.40)	1.40	(1.00–1.96)	1.22	(1.03–1.43)
Current smoker vs. non-current smoker	1.19	(1.02–1.39)	1.52	(1.11–2.08)	1.19	(1.02–1.39)
College− vs. college +	1.05	(0.91–1.20)	0.77	(0.57–1.05)	1.07	(0.94–1.22)
Exercise− vs. +	0.96	(0.82–1.12)	0.93	(0.66–1.31)	0.97	(0.83–1.13)
Blue collar vs. non blue collar	0.63	(0.52–0.77)	0.72	(0.45–1.15)	0.62	(0.52–0.76)
Present illness− vs. +	1.89	(1.64–2.21)	5.00	(3.69–6.78)	1.80	(1.55–2.09)
**Fully adjusted model**						
Workaholism						
Low	1.00		1.00		1.00	
Middle	1.19	(1.02–1.41)	1.76	(1.14–2.73)	1.21	(1.02–1.42)
High	1.38	(1.15–1.69)	3.52	(2.26–5.49)	1.37	(1.13–1.65)
Age						
20–39	1.00		1.00		1.00	
40–49	0.58	(0.48–0.69)	0.56	(0.38–0.82)	0.58	(0.49–0.69)
50–64	0.42	(0.35–0.49)	0.37	(0.26–0.53)	0.42	(0.36–0.50)
Women vs. men	1.15	(1.00–1.32)	1.04	(0.76–1.43)	1.17	(1.02–1.35)
Overweight vs. normal weight	1.19	(1.01–1.41)	1.36	(0.97–1.91)	1.22	(1.04–1.44)
Current smoker vs. non-current smoker	1.20	(1.03–1.40)	1.55	(1.13–2.13)	1.20	(1.03–1.40)
College− vs. college +	1.06	(0.92–1.21)	0.78	(0.57–1.05)	1.08	(0.95–1.24)
Exercise− vs. +	0.96	(0.83–1.13)	0.94	(0.67–1.32)	0.97	(0.83–1.13)
Blue collar vs. non blue collar	0.62	(0.51–0.75)	0.73	(0.45–1.17)	0.61	(0.50–0.74)
Present illness− vs. +	1.88	(1.62–2.19)	4.98	(3.67–6.77)	1.78	(1.54–2.07)
Work hours <60 vs. ≥60 h/wk	0.78	(0.63–0.98)	0.69	(0.42–1.12)	0.77	(0.62–0.97)
Job demand						
Low	1.00		1.00		1.00	
Middle	0.93	(0.78–1.11)	1.17	(0.76–1.81)	0.91	(0.76–1.08)
High	1.05	(0.90–1.23)	1.26	(0.90–1.77)	1.04	(0.88–1.22)
Job control						
High	1.00		1.00		1.00	
Middle	1.06	(0.91–1.23)	0.81	(0.56–1.16)	1.05	(0.90–1.22)
Low	1.17	(0.98–1.40)	1.19	(0.83–1.72)	1.15	(0.96–1.37)
Workplace support						
High	1.00		1.00		1.00	
Middle	0.98	(0.84–1.15)	1.19	(0.82–1.73)	0.99	(0.84–1.15)
Low	0.85	(0.72–1.00)	1.76	(1.22–2.55)	0.84	(0.71–0.99)

OR: Odds Ratio; CI: Confidence Interval.

## Discussion

This study contributes to the literature in that it examines associations that have not been fully explored in previous studies: between workaholism on the one hand and low back pain and sickness absence on the other. We also used a large sample that included workers from various occupations within the tertiary industry. We found a significant association between workaholism on the one hand and psychological ill health, physical ill health (disabling back pain), and sickness absence in general, and mental health-related absence in particular on the other. Furthermore, these associations remained statistically significant even after adjusting for potential confounders including individual characteristics and work-related variables such as weekly work hours, job demand, job control, and workplace support.

The finding of an association between workaholism and psychological ill health is consistent with those of previous studies [6], [7], [10]–[12]. However, most previous studies had a variety of limitation. For instance, previous studies have examined workers from specific occupations or have used samples comprising either women or men alone. In contrast, our sample consisted of employees from various occupations (professionals, technicians, managers, clerks, sales workers, service workers, security workers, and transportation or communications workers) and contained an almost even number of men and women. Therefore, our results indicate that the association between workaholism and psychological health is consistent across several occupations within the tertiary industry.

To our knowledge, this is the first study to find an association specifically between workaholism and LBP by using a standardized definition of LBP [37]. This association remained significant after adjusting for occupation (blue collar vs. non-blue collar). This implies that this association might be independent of work-based physical factors. Nevertheless, the mechanism by which workaholism might lead to disabling LBP is unclear. However, since psychological factors are known to be associated with LBP [38], [39], poor psychological health caused by workaholism may play a role. Given the considerable occupational burden caused by LBP [18]–[21], workaholism needs to be studied further as a risk factor for LBP, along with other psychosocial factors such as job characteristics [26]–[28].

We also observed a significant association between workaholism and sickness absence, even after adjustment for potential confounders. As per the general definition of workaholism, poor psychological and physical health associated with workaholism might result in workers' sickness absence. However, it is possible that the workaholics hesitate to take an absence because they have difficulty in detaching from work, trusting their colleagues, and delegating tasks to others [15]. The present results favor the former possibility. More importantly, higher ORs were found for absence from mental health problems than from other causes. This novel finding can be explained by the characteristics of workaholism. The compulsive style of working based on the tendency for excessive work coupled to an obsession with work [7] may compromise mental health leading to a greater risk of sickness absence. Our current data support the association of workaholism and poor psychological health and the chain of associations needs to be tested in subsequent investigations.

The present study has some limitations. Given the cross-sectional design, causal inferences cannot be made. In addition, the study sample was selected from Internet research volunteers. Our sample may have differed from the population of general Internet users or from a general working population. This may limit the generalizability of our results. Compared with the general population, our sample was also over-representative of people with a higher level of education. Our sample did not include workers from primary or secondary industries. However, the number of workers in the tertiary industry has been increasing, and the tertiary industry is the most predominant industry in Japan [40]. Thus, we believe that our results are reflective of workers living in the urban areas of Japan. However, further studies are necessary in order to examine whether our results are applicable to workers in other industries. The level of workaholism as an independent variable was quantified arbitrarily by tertiles in this study. We did this because the DUWAS does not have a cut-off to determine the level at which employees are regarded as workaholics. Although we examined the association between workaholism and outcomes while carefully adjusting for the effects of relevant factors, some important variables that may explain the associations of interest could not be measured (e.g., alcohol consumption, commuting time, role conflict, role ambiguity, intragroup conflict, or intergroup conflict). The present study used multiple validated scales, resulting in different time frames for responses ranging from the past month to one year. We are not sure how these differences would affect the results, but longer recall times (beyond one year) might have caused more response errors.

In conclusion, workaholism is significantly associated with psychological ill health, disabling back pain, and sickness absence. These results suggest that personal characteristics, as well as work-related environmental factors, need to be addressed when considering the overall well-being of workers.
